# Elucidation of in-vitro anti-inflammatory bioactive compounds isolated from *Jatropha curcas* L. plant root

**DOI:** 10.1186/s12906-015-0528-4

**Published:** 2015-02-05

**Authors:** Ahmad Razi Othman, Norhani Abdullah, Syahida Ahmad, Intan Safinar Ismail, Mohamad Pauzi Zakaria

**Affiliations:** Laboratory of Natural Product, Institute Bioscience, Universiti Putra Malaysia, 43400 Serdang, Selangor Darul Ehsan, Malaysia; Department of Biochemistry, Faculty of Biotechnology and Biomolecular Sciences, Universiti Putra Malaysia, 43400 Serdang, Selangor Darul Ehsan, Malaysia; Institute Of Tropical Agriculture Universiti Putra Malaysia, 43400 UPM Serdang, Selangor Darul Ehsan, Malaysia; Department of Chemistry, Faculty of Science, Universiti Putra Malaysia, 43400 UPM Serdang, Selangor Darul Ehsan, Malaysia; Department of Environmental Sciences, Faculty of Environmental Studies, Universiti Putra Malaysia, 43400 UPM Serdang, Selangor Darul Ehsan, Malaysia

**Keywords:** Plant parts, Hexane extract, Hexadecanoic acid methyl ester, Octadecanoic acid methyl ester, Octadecanoic acid

## Abstract

**Background:**

The *Jatropha curcas* plant or locally known as “Pokok Jarak” has been widely used in traditional medical applications. This plant is used to treat various conditions such as arthritis, gout, jaundice, wound and inflammation. However, the nature of compounds involved has not been well documented. Hence, this study was conducted to investigate the anti-inflammatory activity of different parts of *J. curcas* plant and to identify the active compounds involved.

**Methods:**

In this study, methanol (80%) extraction of four different parts (leaves, fruits, stem and root) of *J. curcas* plant was carried out. Phenolic content of each part was determined by using Folin-Ciocalteau reagent. Gallic acid was used as the phenol standard. Each plant part was screened for anti-inflammatory activity using cultured macrophage RAW 264.7 cells. The active plant part was then partitioned with hexane, chloroform, ethyl acetate and water. Each partition was again screened for anti-inflammatory activity. The active partition was then fractionated using an open column chromatography system. Single spots isolated from column chromatography were assayed for anti-inflammatory and cytotoxicity activities. Spots that showed activity were subjected to gas chromatography mass spectrophotometry (GC-MS) analysis for identification of active metabolites.

**Results:**

The hexane partition from root extract showed the highest anti-inflammatory activity. However, it also showed high cytotoxicity towards RAW 264.7 cells at 1 mg/mL. Fractionation process using column chromatography showed five spots. Two spots labeled as H-4 and H-5 possessed anti-inflammatory activity, without cytotoxicity activity. Analysis of both spots by GC-MS showed the presence of hexadecanoic acid methyl ester, octadecanoic acid methyl ester and octadecanoic acid.

**Conclusion:**

This finding suggests that hexadecanoic acid methyl ester, octadecanoic acid methyl ester and octadecanoic acid could be responsible for the anti-inflammatory activity of the *J. curcas* root extract.

## Background

*Jatropha curcas* Linn. (family Euphorbiaceae) is a multipurpose plant, brought by the Portuguese sailor from Mexico to the Asian region around the 16^th^ century to be used as shade tree and live fences for crops protection [1]. However, the plant was discovered to possess healing properties and for decades it has been used to treat arthritis, gout, jaundice and inflammation in countries such as Peru, Brazil, Mexico and Egypt [2]. Currently, increasing scientific interest focuses on the importance of bioactive compounds derived from plants including *J. curcas.*

A group of researchers from Malaysia reported that Jatropha poisoning occurred mainly in children due to accidental ingestion of the fruits of the plant [3]. Similar cases were also reported in India in 2003 [4]. However, to date, there is no report on the lethal effect of Jatropha in human [5]. Nevertheless, several cases involving death were reported in ruminant associated with Jatropha consumption during the dry season [6].

It has been shown that the root of *J. curcas* exerts a significant anti-inflammatory effect in bioassay studies [7,8]. Unfortunately, the root of *J. curcas* does not only exert beneficial anti-inflammatory effect, but it also shows toxic effects on humans, animals and cell lines. A study conducted in Brazil found that the tribes using *J. curcas* roots as traditional medicine were advised to take extra precaution due to the toxicity of the plant [2]. These results indicated that the *J. curcas* root contains compounds with anti-inflammatory as well as cytotoxic activities.

Thus, the main aim of this study was to elucidate the nature of bioactive compounds with anti-inflammatory and cytotoxic activities present in *J. curcas* plant.

## Methods

### Sample collection

*Jatropha curcas* L. plant aged approximately 5 years was collected from Farm 2, Faculty of Agriculture, Universiti Putra Malaysia (UPM) and verified by the botanist, Dr. Shamsul Khamis at the Institute of Bioscience, UPM. The plant was deposited in the Phytomedicinal Herbarium, Institute of Bioscience, UPM with voucher number (SK1764/2010). Three plants were bought to the laboratory and distributed into leaves, fruits, stems and roots.

### Preparation of plant crude extract

All plant parts were cut into small pieces (2 mm to 5 mm), dried using a freeze drier and ground to a powder form. Subsequently, 10 mg of dried powdered samples were transferred into the 250 mL bottle. Samples were then soaked in 200 mL 80% methanol for 24 hours. The samples were filtered through Whatman filter paper No. 1 and the filtrate was kept in 1 L bottle and stored at 4°C. The residue was re-extracted with the same volume of 80% methanol every day for five days until the solvent color became clear. All filtrates were pooled and dried using a rotary evaporator at 40°C with constant pressure. Dried extracts were labeled as crude extracts and stored at −20°C for screening of anti-inflammatory activity [7].

### Liquid – liquid partition

The dried methanolic extract (6.4 g) was dissolved in 1 L of double distilled water before undergoing liquid-liquid partition. Three types of solvents (hexane, chloroform, ethyl acetate) with different polarities were used as described by Rajbir et al. [9]. Each partition was conducted three times and the eluent was pooled and dried using rotary evaporator. Dry weight of each partition was recorded as total yield.

### Phenolic content

Phenolic content of crude extract was determined by using Folin Ciocalteau method, using gallic acid as the standard. Twenty uL of crude extract (1 g/mL) were added into 1.58 mL of distilled deionized water. A 100 uL of Folin Ciocalteau reagent (5%) was added and mixed thoroughly and incubated for 8 minutes at room temperature. After that, 300 uL of concentrated sodium carbonate (Na_2_CO_3_) were added and incubated for 2 hours. The absorbance was read at 765 nm using a spectrophotometer (Labomed, inc. model Spectro 23). A gallic acid standard (0–0.05 mg) was prepared accordingly. The phenolic content was expressed as milligram of gallic acid equivalent (GAE) per gram of dried samples (mg GAE/g dw).

### Anti-inflammatory and cytotoxicity assays

Murine monocytic macrophage cell line RAW 264.7 purchased from American Type Culture Collection (ATCC) was cultured in Dulbecco’s Modified Eagle Media (DMEM) with 4 mM L-Glutamine, 45 g/L glucose, 1 mM sodium pyruvate and 10% of fetal bovine serum (FBS). The cells were incubated in 50 mL cell culture flask placed in CO_2_ incubator with 5% of CO_2_ at 37°C. RAW 264.7 cells were seeded in a 96-well microplate at 1x10^6^ cell/mL. Seeded cells were incubated in 5% CO_2_ at 37°C for 2 to 3 hours. One mg/mL of samples was added to seeded cells. Cells were then stimulated with 100 U/mL of interferon-gamma (IFN-γ) and 5 ug/mL of lipopolysacharide (LPS) from *Escherichia coli* strain 055:B5. The cells were incubated in 5% CO_2_ at 37°C for 18 hours. The nitric oxide concentration produced by RAW 264.7 cell was determined by Griess assay. N-nitro-L-arginine-methyl ester (L-NAME) as iNOS inhibitor was used as a positive control at concentration of 250 μM. The cytotoxicity activity of sample extract was determined using MTT (3-(4,5-dimethylthiazol-2-yl)-2,5-diphenyltetrazolium bromide) assay [10].

### Column chromatography analysis

Five mL of mini preparative columns were prepared using silica gel 60 with 70–230 Mesh (ASTM) slurry in 100% hexane. The column was then prewashed with hexane: ethyl acetate (EtOAc) solvent at ratio 7:3. One mL of fraction was loaded into the column and immediately eluted with 3 mL of hexane: EtOAc (7:3) solution, followed by 20 mL of hexane: EtOAc solution (6:4). One mL of eluent was collected in 1.5 mL microcentrifuge tube in series. Each eluent was analyzed using analytical thin layer chromatography (TLC) plate.

Thin layer chromatography plate was prepared by coating a plastic plate with silica gel 60 F245 (0.25 mm thick and 7.5 cm long). The developer solvent was hexane: EtOAc with a ratio 6:4. A developed TLC plate was then viewed under UV at wavelength 254 nm and 366 nm. Spots with similar R_f_ values were pooled and air dried for anti-inflammatory assay and metabolites identification.

### Gas chromatography mass spectrometry (GC-MS)

Hexane partition of root and spot samples from TLC plates were analysed for bioactive compounds by using GC-MS QP2010 Plus SHIMADZU with SGE BPX5 column (30 m × 0.25 mm, I.D × 0.25 μm). Oven temperature was set at 50°C to 300°C at 10°C/min. Injection temperature was set at 250°C using a splitless mode. Helium gas (99.9%) was used as the carrier. Total GC-MS running time was 35 minutes. All peak areas were compared with the database in the GC-MS library version NIST 08-S.

### Statistical analysis

All data were subjected to one-way analysis of variance (ANOVA). Treatment means were compared using Tukey’s multiple comparison tests. Statistic software Graphpad Prism 5.0 (Graphpad Software Inc., San Diego, CA) was used for all statistical analyses.

## Results

### Phenolic content

The levels of phenolic compounds in different parts of *J. curcas* are shown in Table 1. Among the parts, leaves showed the highest value, followed by roots, fruits and stem. Differences among plant parts were significant (P < 0.05). The value for leaves, roots, fruits and stem were 1.33 mg GAE/g dw, 1.11 mg GAE/g dw, 0.51 mg GAE/g dw and 0.11 mg GAE/g dw respectively. Phenolic content for *J. curcas* root partitions is shown in Table 2. The phenolic content in water fraction was significantly higher (P < 0.05) than the other fractions. The values for water, chloroform, ethyl acetate and hexane were 0.69 mg GAE/g dw, 0.15 mg GAE/g dw, 0.15 mg GAE/g dw and 0.13 mg GAE/g dw, respectively.

**Table 1:** **Phenolic contents of different parts of** ***J. curcas***

**Plant parts**	**Phenolic (mg GAE/g dw)** ^*^
Leaves	1.33 ± 0.013^a^
Root	1.11 ± 0.008^b^
Fruits	0.51 ± 0.017^c^
Stem	0.11 ± 0.003^d^

*Dry weight of extract.

Means ± standard deviation with different superscripts indicate significant difference.

(P < 0.05).

**Table 2:** **Phenolic content of** ***J. curcas*** **root in different solvents**

**Fractions**	**Phenolic (mg GAE/g dw)** ^*^
Water	0.69 ± 0.009^a^
Chloroform	0.15 ± 0.004^b^
Ethyl Acetate	0.15 ± 0.004^b^
Hexane	0.11 ± 0.004^b^

*Dry weight of extract.

Means ± standard deviation with different superscripts indicate significant difference.

(P < 0.05).

### Anti-inflammatory screening of crude extract

**Figure 1:**
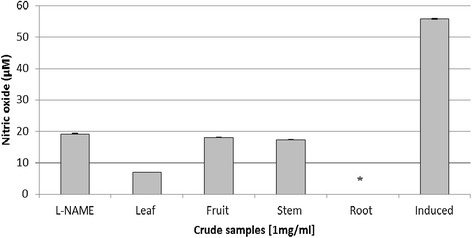
**Anti-inflammatory activity of methanolic extract in RAW 264.7 macrophage cells.** RAW 264.7 cells were induced using LPS/IFN-γ. L-NAME (250 μM) and induced cell represented a positive and negative control respectively. Each histogram represents a mean of three replicates with error bar representing the standard deviation. *Indicates significant difference (P < 0.05) compared to the positive control.

**Figure 2:**
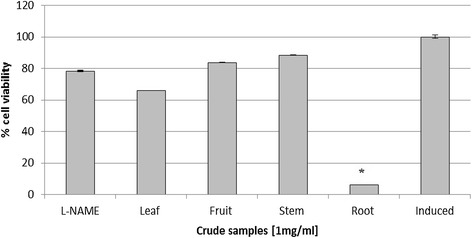
**Cytotoxic activity of methanolic extract of different parts of** ***J. curcas*** **plant using MTT assay.** L-NAME (250 μM) and induced cell represented positive and negative control respectively. Each histogram represents a mean of three replicates with error bar representing the standard deviation. *Indicates significant difference (P < 0.05) compared to the positive control.

Anti-inflammatory activity was evaluated using the methanolic extract from different parts of *J. curcas*. Crude extract from root gave 100% of inhibition towards nitric oxide production (Figure 1). Cytotoxicity test using MTT assay showed that crude extract from root gave the highest inhibition towards cell growth. Almost 95% of the cell growth was inhibited by the crude extract (Figure 2).

### Liquid-liquid partition yield

**Figure 3:**
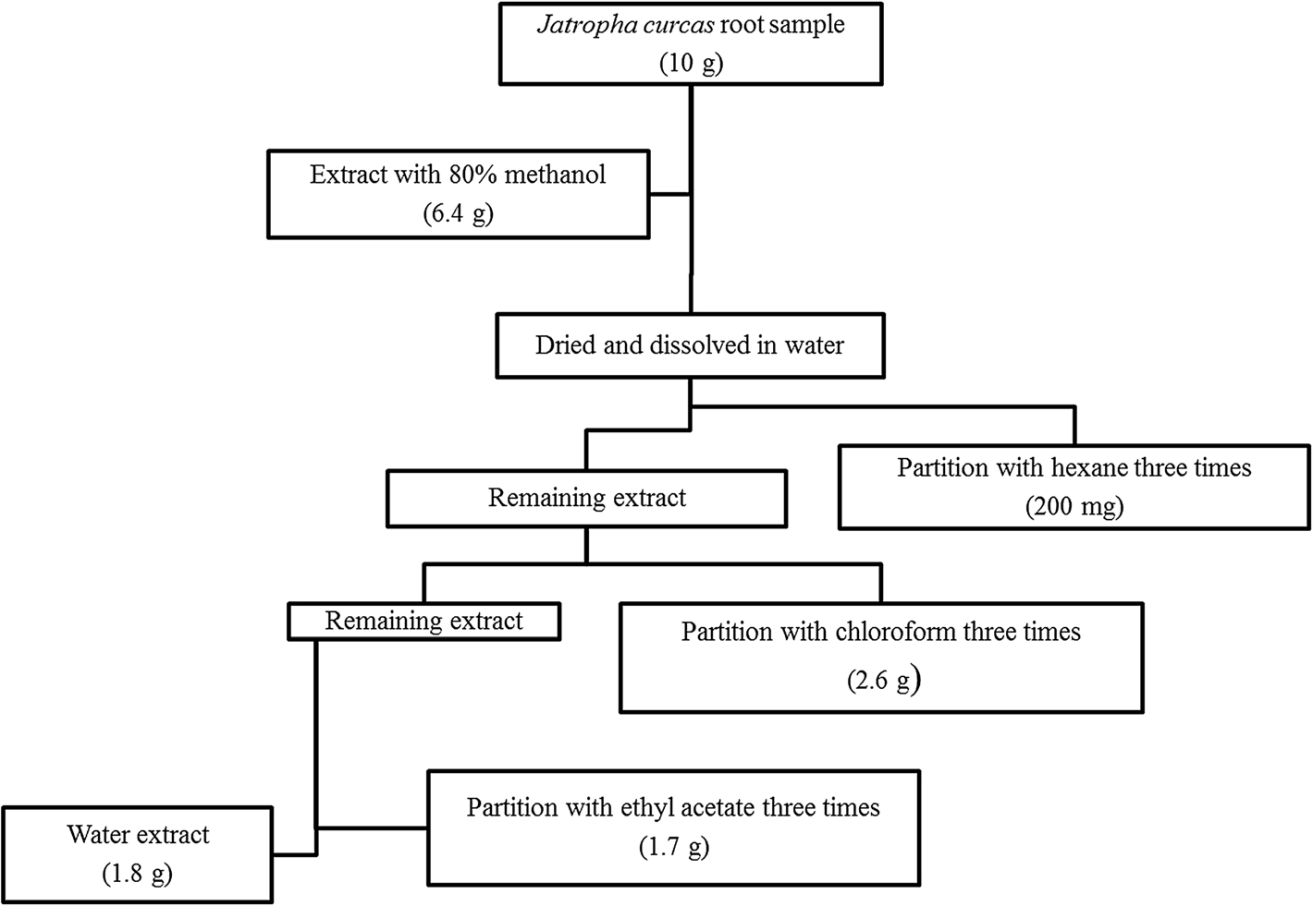
**A flowchart of a liquid-liquid partition step of a crude extract from** ***J. curcas*** **root.** Dry weight of samples obtained is stated in the flowchart.

Approximately 6.4 g of crude extract was obtained from 10 g root sample. Liquid-liquid partition produced extract of hexane, chloroform, ethyl acetate and water at 200 mg, 2.6 g, 1.7 g and 1.8 g, respectively (Figure 3).

### Anti-inflammatory screening of root extract partition

**Figure 4:**
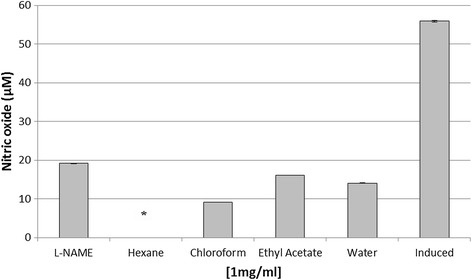
**Anti-inflammatory activity of different solvent partitions.** Crude extract from root was subjected to liquid-liquid fractionation process. Each partition was subjected to anti-inflammatory test. L-NAME (250 μM) and induced cell represented a positive and negative control respectively. Each histogram represents a mean of three replicates with error bar representing the standard deviation. *Indicates significant difference (P < 0.05) compared to the positive control.

**Figure 5:**
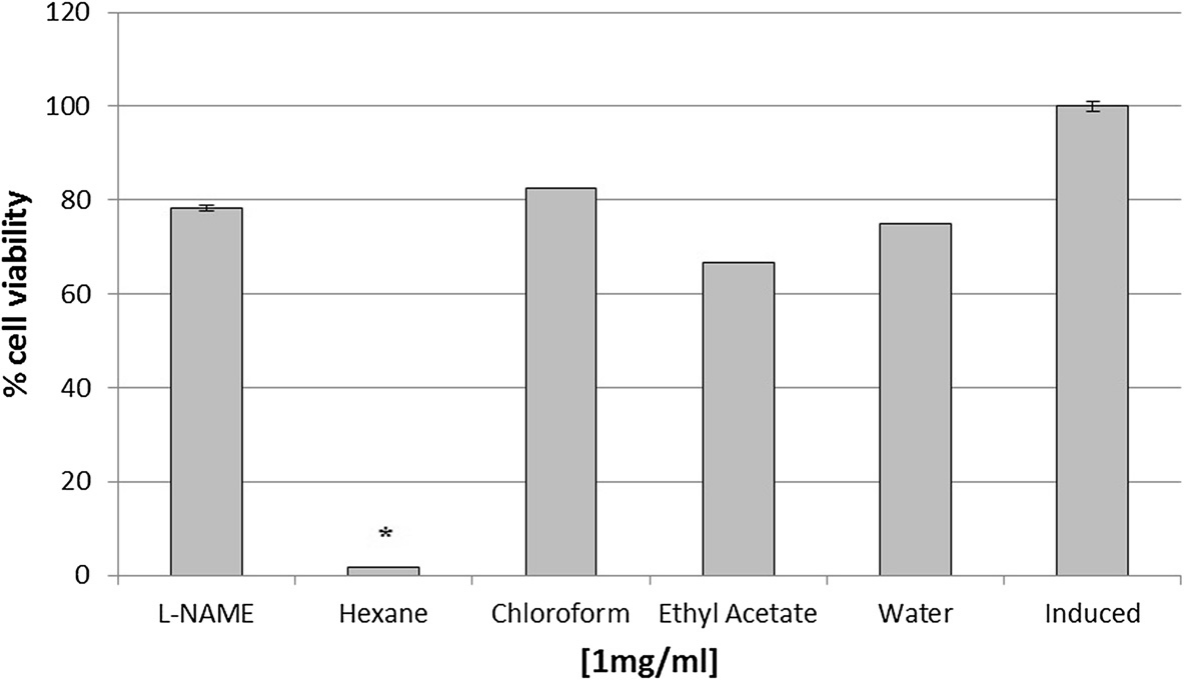
**Cytotoxic activity of different solvent partitions of root by MTT assay.** L-NAME (250 μM) and induced cell represented a positive and negative control respectively. Each histogram represents a mean of three replicates with error bar representing the standard deviation. *Indicates significant difference (P < 0.05) compared to the positive control.

Anti-inflammatory activity was carried out using different solvent partitions of *J. curcas* root. Among the four partitions tested, hexane fraction showed the highest inhibition followed by chloroform, water and ethyl acetate with values significantly different (P < 0.05) compared to the positive control (Figure 4). A cytotoxicity test by MTT assay showed hexane partition produced the highest inhibition towards cell growth, whereas other partitions exhibited no cytotoxicity effect (Figure 5). Analysis of the hexane partition by GC-MS indicated the presence of various compounds mainly polycyclic aromatic hydrocarbons and fatty acid derivatives (Table 3). A number of these compounds were terpenoids such as 17 alpha-hydroxypregnenolone, bicyclo[4.2.0]oct-1-ene, 2-methyl-7-exo-phenyl and gamma-sitosterol.

**Table 3:** **The major compounds detected in hexane fraction by GC-MS analysis**

**No**	**Compound name**	**Percentage area**
1	9-Octadecanoic acid, methyl ester	2.22
2	Bicyclo[4.2.0]oct-1-ene, 2-methyl-7-exo-phenyl*	10.79
3	5-[2-(4,5,5-Trimethyl-cyclopent-1-enyl)ethylidene]pyrimidine-2,4,6(1H,3H,5H)-trione*	8.94
4	9-Methyl-S-octahydrophenanathracene*	2.69
5	17.alpha-Hydroxypregnenolone*	7.57
6	Delta.-Selinene*	2.65
7	4a.alpha, 4b.beta-Gibbane-1.alpha10. beta-dicarboxilic acid*	2.72
8	2,4-Bis(dimethylbenzyl)-6-t-butylphenol*	3.55
9	Phenol, 2,4-bis(1-methyl-1-phenyl)*	2.42
10	1,2-Benzenedicarboxilic acid, mono(2-ethylhexyl) ester*	9.95
11	Decanedioic acid,bis(2-ethylhexyl) ester	6.69
12	Gamma-Sitosterol*	2.79

*polycyclic aromatic hydrocarbon derivatives.

### Anti-inflammatory assay of isolated compounds

**Figure 6:**
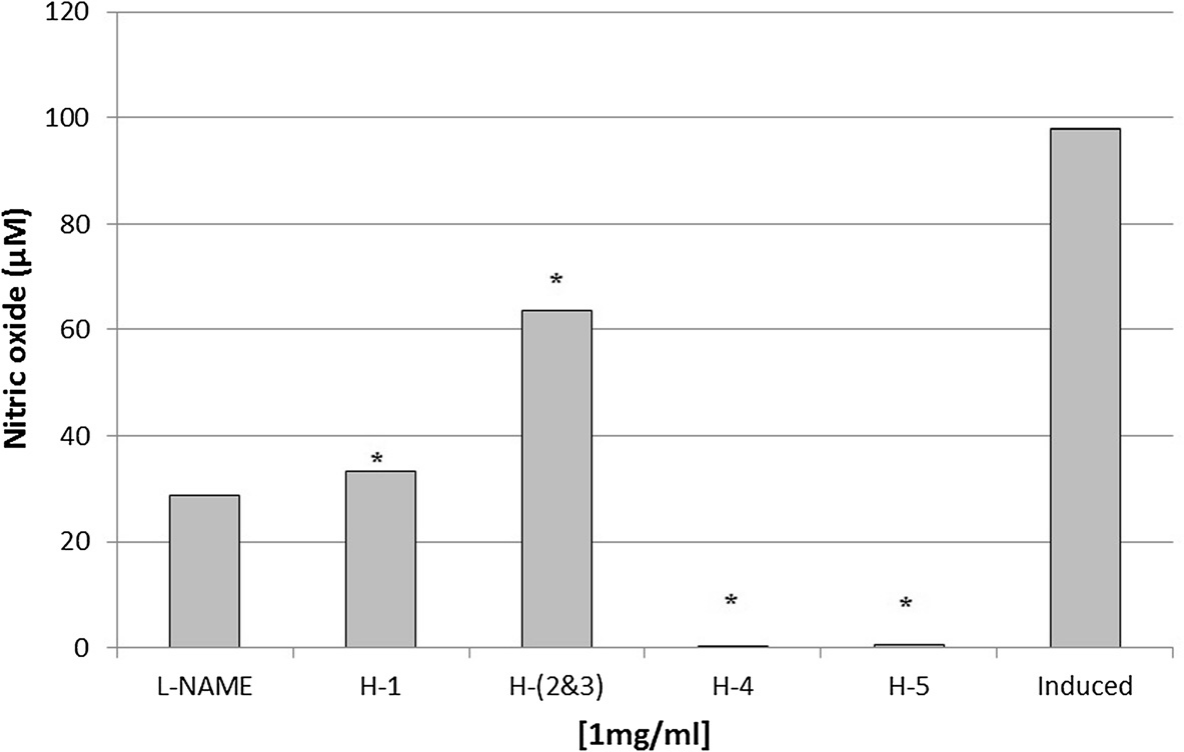
**Anti-inflammatory activity of different spots isolated from hexane fraction.** Spot H-(2&3) consisted of two partially overlapped spots. Spot H-4 and H-5 showed high anti-inflammatory activity. L-NAME (250 μM) and induced cell represented a positive and negative control respectively. Each histogram represents a mean of three replicates with error bar representing the standard deviation. *Indicates significant difference (P < 0.05) compared to the positive control.

**Figure 7:**
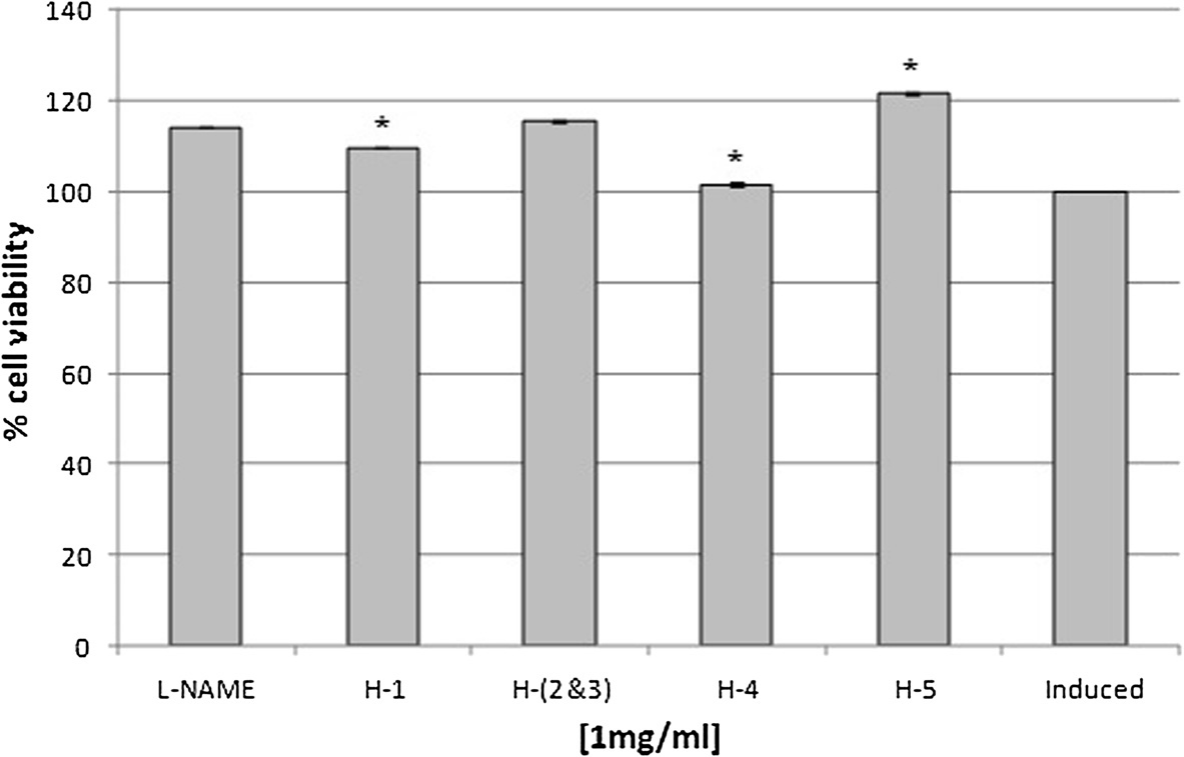
**Cytotoxic activity of different spots isolated from hexane partition using MTT assay.** All spots showed no toxicity effect towards RAW 264.7 cells growth. L-NAME (250 μM) and induced cell represented a positive and negative control respectively. Each histogram represents a mean of three replicates with error bar representing the standard deviation. *Indicates significant difference (P < 0.05) compared to the positive control.

Hexane fraction was chosen as it showed both anti-inflammatory and cytotoxicity activities for column chromatography analysis using silica gel (0.06 – 0.2 mm/70–230 Mesh ASTM). The separation produced five single spots, labeled as H-1 to H-5. Spot H-4 and H-5 showed strong inhibition towards nitric oxide production by RAW 264.7 murine macrophage cells, while spots labeled as H-1 and H-(2&3) showed lower inhibition when compared to the positive control (Figure 6). Cytotoxicity test using MTT assay showed no inhibition towards cell growth for all spots (Figure 7).

### GC-MS analysis of active compounds

**Figure 8:**
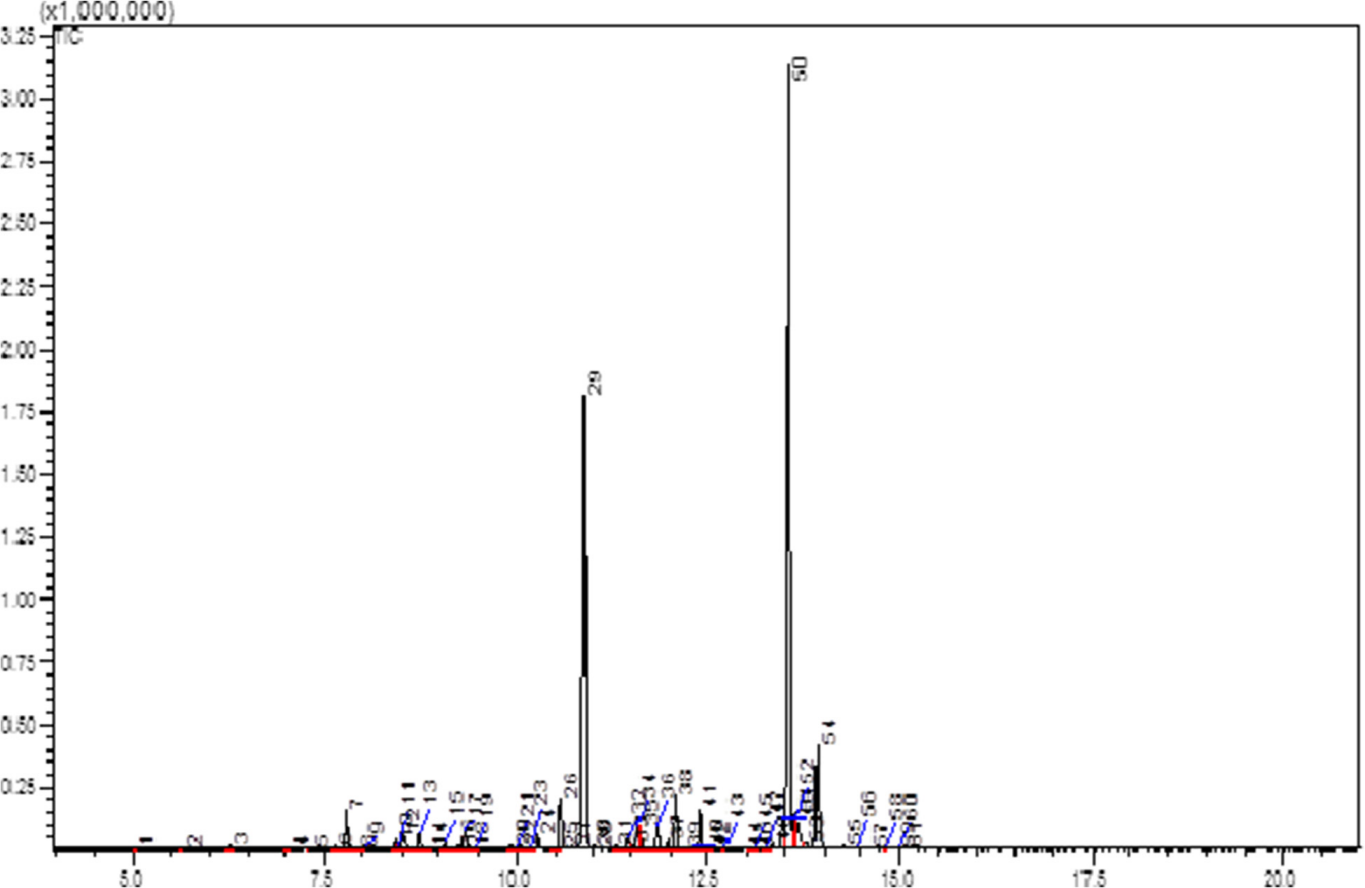
**GC-MS spectrum of H-4 spot from hexane fraction.** Two major peaks were identified. Peak number 29 at 10.875 minutes was identified as hexadecanoic acid methyl ester, while peak number 50 at 13.559 minutes was identified as 9-octadecanoic acid methyl ester.

**Figure 9:**
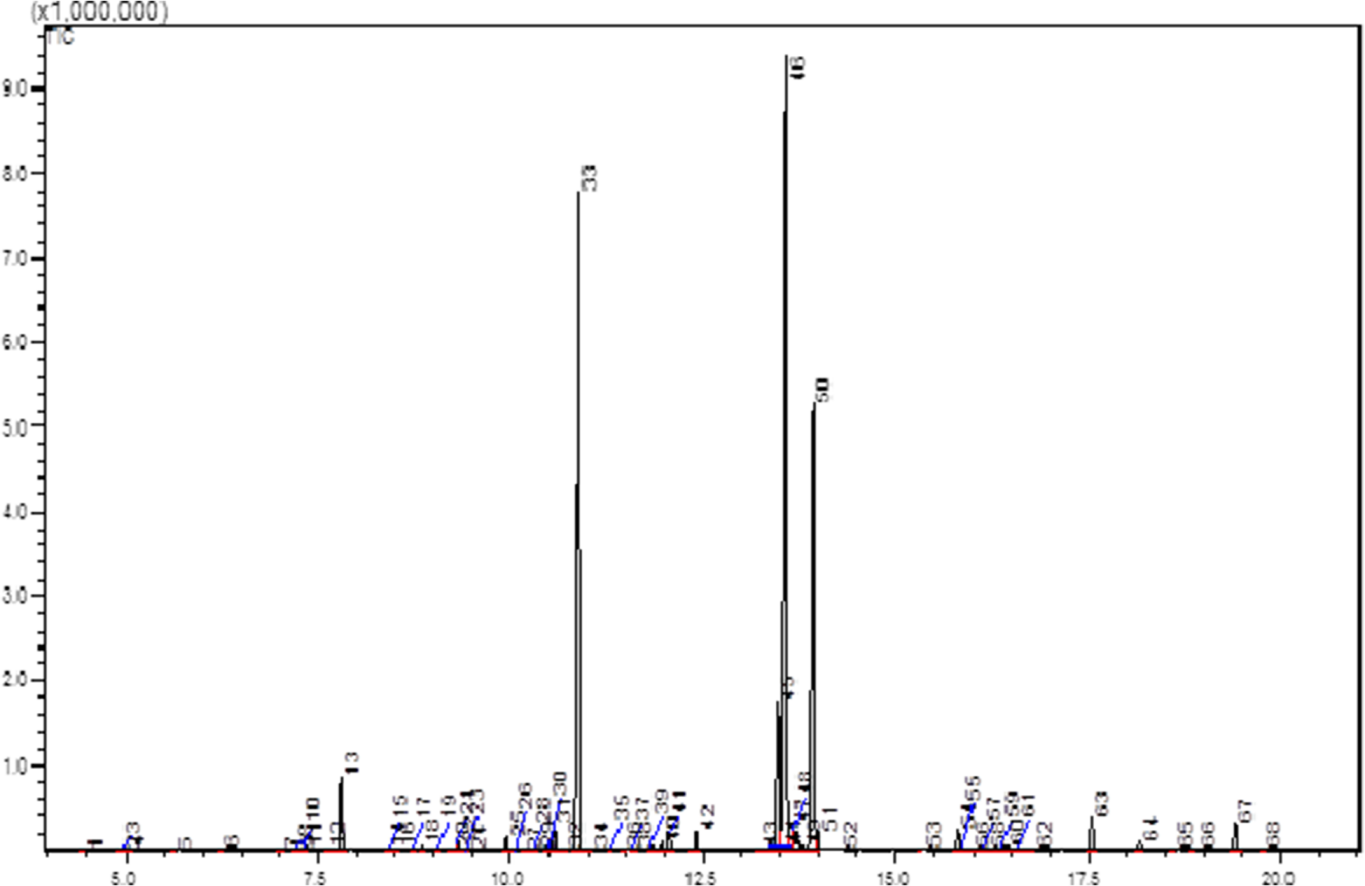
**GC-MS spectrum of H-5 spot from hexane fraction.** Three major peaks were identified. Peak number 33 at 10.888 minutes was identified as hexadecanoic acid methyl ester, peak number 46 at 13.574 minutes was identified as 9-octadecanoic acid methyl ester and peak number 50 at 13.930 minutes was identified as octadecanoic acid.

The active compounds present in the spots H-4 and H-5 were analyzed using GC-MS and identified based on the database in the GC-MS library. The major groups of these spots were fatty acids. Phenolic compounds also exist as a minor component in this particular fraction. The H-4 spot showed two major peaks (29 and 50) at retention times of 10.875 and 13.559 minutes and were identified as hexadecanoic acid methyl ester and 9-octadecanoic acid methyl ester, respectively (Figure 8). The H-5 spot showed three major peaks (33, 46 and 50) and identified as hexadecanoic acid methyl ester, 9-octadecanoic acid methyl ester and octadecanoic acid, respectively (Figure 9).

## Discussion

Among the plant parts of *J. curcas*, leaves showed the highest amount of phenolics expressed in milligram of gallic acid equivalent (mgGAE/g dw), followed by root, fruits and stem. Similarly, it was reported that leaves of *J. curcas* contained the highest amount of phenolic compounds, but not for roots and stem part where phenolic content for stem was higher than that of roots [7].

It has been reported that phenolic compounds with various biological activities are commonly produced by plants. Phenolic compounds extracted from *Gastrodia elata* root showed an antioxidant property [11], while phenolic compounds extracted from *Nymphaea mexicana Zucc* showed an anti-inflammatory property towards induced RAW 264.7 macrophage cells [12]. It has been suggested that the anti-inflammatory property of the phenolic compounds was due to their scavenging activities on the reactive oxygen species or reactive nitrogen species [13].

The effectiveness of *J. curcas* root in anti-inflammatory activity had been reported by in vivo studies. It has been shown [8] that the root extract could reduce formalin induced paw edema in rat. Later, Nayak and Patel [14] compared root, stem and leaf extracts of *J. curcas* in anti-inflammatory activity study towards carrageenan-induced rat paw edema. Groups of albino rats were injected by inflammation inducer (carrageenan) and the levels of inflammation mediators (histamine, serotonin and kinin) were monitored. Their results showed that root extract inhibited histamine, serotonin and kinin released up to 70% - 80% compared to leaf (50% - 55%) and stem (40% - 45%). However, these studies did not indicate the nature of compounds involved in the anti-inflammatory activity.

It was also observed that, root extract also exhibited a high cytotoxicity activity towards RAW 264.7 cells growth. A similar finding was also reported [7] where a series of different concentrations of methanolic extract of *J. curcas* root showed growth inhibition of cells in a dose-dependent manner. At this point, it was not certain whether the anti-inflammatory activity was due to cell death or genuine inhibitory effect of the compounds present in the extract or that the extract contained compounds with anti-inflammatory as well as cytotoxicity activities. It was also interesting to note that, in the present study, methanolic extract of *J. curcas* roots which contained lower phenolic compounds compared to that of leaves extract, showed the highest anti-inflammatory activity towards RAW 264.7 cells. This indicated that root extract may contain non-phenolic compounds with anti-inflammatory activity.

Further purification conducted by liquid-liquid partition separated the root crude extract into four fractions based on the different solvent polarities. Among the four solvents, water fraction showed the highest phenolic content (0.69 mg GAE/g dw), while chloroform, ethyl acetate and hexane fractions showed similar levels (0.13 - 0.15 mg GAE/g dw). Although all partitions showed an inhibition towards nitric oxide production in RAW 264.7 cells, the hexane fraction showed the highest inhibitory activity. Again, this finding indicated that other non-phenolic compounds may be involved in the anti-inflammatory activity. Previous studies had reported the anti-inflammatory activity of the non-polar solvent extracts from various plants species. For example, ethyl acetate fraction of *Ochna squarrosa* L. showed a higher anti-inflammatory activity when compared to diclofenac sodium [15]. Another report showed that the anti-inflammatory activity of the hexane extract of *Alchornea cordifolia* was better than indomethacin in an in vivo study [16]. Several other reports also observed that a non-polar extract from *Urtica dioica* L. and *Acalypha indica* exhibited an anti-inflammatory property [17,18]. Their results augur well with the findings of the present study where hexane fraction showed the strongest inhibition, indicating non-polar compounds were involved.

However, the hexane fraction was also found to be toxic towards RAW 264.7 cells. GC-MS analysis of the hexane fraction showed the presence of several compounds belonging to the terpenoid groups such as 5-[2-(4,5,5-trimethyl-cyclopent-1-enyl)ethylidene]pyrimidine-2,4,6(1H,3H,5H)-trione;1,2-benzenedicarboxilic acid, mono(2-ethylhexyl) ester and bicyclo[4.2.0]oct-1-ene, 2-methyl-7-exo-phenyl. Terpenoids have been claimed in several reports to have cytotoxicity effects towards animal cells lines. Terpenoid compounds commonly found abundant in plants exhibited anti-cancer property by killing the cancer cells [19]. A report using *J. curcas* seed kernel extract showed that the extract which contained terpenoid compounds inhibited breast cancer cells (MCF-7) and cervical cancer (HeLa) growth in a dose-dependent manner [20]. Other studies have also reported the cytotoxicity effect of terpenoid compounds on cancer as well as normal cells growth. A cytotoxicity test of terpenoid compounds showed total growth inhibition of normal green monkey kidney cells (VERO) at 0.21 μg.mL^−1^ [21].

Further separation procedures by the open system column chromatography produced five spots, which were non-toxic towards RAW 264.7 cell lines. However, only two spots (H-4, H-5) gave positive results towards anti-inflammatory activity. This indicated that the cytotoxic compounds present in hexane extract were successfully separated from the anti-inflammatory metabolites. The separation was probably due to terpenoids being un-eluted as they bound strongly to the silica gel column because of their polarities.

Identification of the active compounds present in spots H-4 and H-5 by GC-MS revealed the presence of long chain fatty acids as the major compounds. The three major fatty acids identified by GC-MS were hexadecanoic acid methyl ester, 9-octadecanoic acid methyl ester and octadecanoic acid. Several reports have claimed that saturated fatty acids are involved in regulating anti-inflammatory pathway [22,23]. An enzyme kinetic study [23] proved that saturated fatty acid acted as a competitive inhibitor towards phospholipase A_2_, an enzyme involved in catalyzing the release of arachidonic acid, a precursor for the synthesis of inflammatory cytokine at *sn*-2 position of membrane phospholipid [24,25]. The absence of terpenoid compounds in the active spots strongly supported the above suggestion that the cytotoxicity effect was due to the presence of terpenoid compounds in the samples.

## Conclusion

In conclusion, the present study demonstrated that among the different parts of *J. curcas* plant, the root extract showed the highest anti-inflammatory activity in RAW 264.7 macrophage cell lines. However, the hexane fraction of *J. curcas* root extract possessed both anti-inflammatory and cytotoxicity activities. The non-polar compounds present in the hexane fraction analyzed by GC-MS consisted of terpenoids which may contribute to the cytotoxicity activity. A further purification step by column chromatography of the hexane fraction produced non-cytotoxic metabolites with anti-inflammatory activity. These metabolites analyzed by GC-MS were identified as hexadecanoic acid methyl ester, 9-octadecanoic acid methyl ester and octadecanoic acid.

## Abbreviations

GC-MS: Gas chromatography mass spectrophotometer
DMEM: Dubelco’s modified eagle media
FBS: Fetal bovine serum
IFN-γ: Interferon-gamma
LPS: Lipopolysaccharide
L-NAME: N-nitro-L-arginine-methyl ester
iNOS: Inducible nitric oxide synthase
MTT: 3-(4,5-dimethylthiazol-2-yl)-2,5-diphenyltetrazolium bromide
EtOAc: Ethyl acetate
TLC: Thin layer chromatography
UV: UltraViolet
GAE: Gallic acid equivalent
